# Endoplasmic reticulum stress enhances the antigen-specific T cell immune responses and therapeutic antitumor effects generated by therapeutic HPV vaccines

**DOI:** 10.1186/s12929-019-0536-7

**Published:** 2019-05-27

**Authors:** Sung Yong Lee, Jee Youn Oh, Tae Heung Kang, Hyun Seock Shin, Max A. Cheng, Emily Farmer, T.-C. Wu, Chien-Fu Hung

**Affiliations:** 1 Department of Respiratory & Critical Care Medicine, Korea University Medical Center, 73 Inchon-ro, Seongbuk-gu, Seoul, 02841 South Korea; 2 Department of Immunology, Konkuk University Medical Center, 120 Neungdong-ro, Gwangjin-gu, Seoul, 05029 South Korea; 3 Department of Pathology, Korea University College of Medicine, 73 Inchon-ro, Seongbuk-gu, Seoul, 02841 South Korea; 4 Department of Pathology, Johns Hopkins Medical Institutes, 1550 Orleans Street, Cancer Research Building II, Baltimore, MD 21287 USA; 5 Department of Obstetrics & Gynecology, Johns Hopkins Medical Institutes, 1550 Orleans Street, Cancer Research Building II, Baltimore, MD 21287 USA; 6 Department of Molecular Microbiology & Immunology, Johns Hopkins Medical Institutes, 1550 Orleans Street, Cancer Research Building II, Baltimore, MD 21287 USA; 7 Department of Oncology, Johns Hopkins University School of Medicine, CRB II Room 307, 1550 Orleans Street, Baltimore, MD 21287 USA

**Keywords:** HPV, HPV16, Therapeutic HPV vaccine, pcDNA3-CRT/E7, 3-bromopyruvate, Endoplasmic reticulum stress, CHOP, GRP78, CD8 T cells

## Abstract

**Background:**

Endoplasmic reticulum stress has a profound effect on cancer cell proliferation and survival, and also has the capacity to activate cells of the adaptive immune system. Multimodal treatment methods that utilize and combine conventional cancer therapies with antigen-specific immunotherapies have emerged as promising approaches for the treatment and control of cancer. However, it is not well known whether endoplasmic reticulum stress-inducing agents can influence the efficacy of tumor antigen-targeting vaccines.

**Methods:**

In the past, we developed a therapeutic human papillomavirus (HPV) DNA vaccine that encodes for calreticulin (CRT) linked to the HPV16 E7 antigen (CRT/E7). In this study, we utilize the CRT/E7 and further encode for an endoplasmic reticulum (ER) stress-inducing agent, 3-bromopyruvate (3-BrPA), in a preclinical model, by harnessing its potential to enhance HPV16 E7-specific CD8+ T cell immune responses as well as antitumor effects against E7-expressing tumors (TC-1 cells). E7-specific CD8+ T cells were added to evaluate the cytotoxicity of luciferase-expressing TC-1 tumor cells treated with 3-BrPA in vitro, as measured with an IVIS Luminescence Imaging System. We also determined the levels of ER stress markers in 3-BrPA-treated TC-1 cells. TC-1 tumor-bearing mice were treated with either 3-BrPA (10 mg/kg, intraperitoneal injection) and/or CRT/E7 DNA vaccine (30 μg/mouse).

**Results:**

Treatment of E7-expressing TC-1 tumor cells with 3-BrPA induced significantly higher in vitro cytotoxicity and resulted in upregulation of endoplasmic reticulum stress markers (CHOP and GRP78). More importantly, combination treatment of 3-BrPA and the CRT/E7 DNA vaccine led to improved antigen-specific CD8+ T cell immune responses as well as therapeutic antitumor effects in TC-1 tumor-bearing mice.

**Conclusions:**

Our data indicate that 3-BrPA can enhance therapeutic HPV vaccine potency in generating improved antigen-specific immune responses and antitumor effects. These findings have important implications for future clinical translation and provide novel strategies for the treatment of HPV-associated diseases.

## Background

Recently, treatments such as immune checkpoint inhibitors have shown a strong therapeutic effect in several cancer treatments, while other immunotherapies are gradually receiving more attention in cancer therapy [2, 13, 28]. PD-1/PD-L1 and CTLA-4 monoclonal antibodies have significantly improved the survival of metastatic melanoma patients, producing durable responses in about 20–40% of patients when used as monotherapies, and in up to 60% of the combination therapy [24, 32]. PD-1/PD-L1 blockades have achieved impressive clinical results in advanced NSCLC patients, where it is now being investigated in combination with CTLA-4 blockade [11, 22]. Unfortunately, there is concern about the limitations of immune checkpoint therapy, including response heterogeneity, as many patients who receive checkpoint therapy did not demonstrate strong clinical efficacy [28]. Therefore, immunotherapy extends beyond immune checkpoint therapy by employing other immune stimulating strategies such as tumor-associated antigen expressing vaccine therapy and immunogenic cell death, inducing agents that target malignant cells and promote their destruction. DNA vaccines emerge as a practical and attractive approach with great potential to translate to the clinical setting. DNA vaccines are known to be highly stable, and their safety profile has been well-established [3, 18]. DNA vaccines are also easy to prepare and produce at high purity, and allow for multiple administrations [3]. Practically, they are more cost-effective and transportable when compared to other vaccines, such as recombinant protein, tumor cell, or viral vector vaccines. Genes in DNA vaccines can also be designed to encode different antigens as well as various other immunomodulatory molecules to manipulate the resulting immune responses. Despite all the advantages, DNA vaccines have had limited success in producing therapeutic effects against most cancers, due to poor immunogenicity [3]. Therefore, additional strategies are required in an attempt to enhance DNA vaccine potency.

The endoplasmic reticulum (ER) is a specialized organelle that plays a central role in the biosynthesis, correct protein folding, and post-translational modification of secretory and membrane proteins [30]. In cancer, ER stress has the capacity to activate cells of the adaptive immune system [1]. ER stress alone is sufficient to trigger systemic inflammation by proteolytic activation of the transcription factor cyclic-AMP-responsive-element-binding protein H (CREBH) at the ER membrane [31, 35]. ER stress-mediated cell surface presentation of calreticulin (CRT) has emerged as a damage-associated molecular pattern (DAMP) of potential importance in cancer [8, 21]. It is not well-known whether ER stress-inducing agents can influence the efficacy of tumor antigen-targeting vaccines. In our current study, we studied the improvement in efficacy of the CRT/E7 DNA vaccine using the ER stress-inducing agent 3-bromopyruvate (3-BrPA).

## Materials and methods

### Mice

Six-to-8-week-old female C57BL/6 mice were purchased from the National Cancer Institute (Frederick, MD) and housed in the Oncology Center Animal Facility at the Johns Hopkins Medical Institutes (Baltimore, MD). All animal procedures were performed according to approved protocols and in accordance with recommendations for the proper use and care of laboratory animals. To ensure that animal discomfort, distress, pain, and injury were kept to a minimum, a maximum of 5 mice were housed in the same cage. All animals were monitored on a daily basis using Johns Hopkins Medical Institutions Animal Care and Use Committee guidelines.

### Reagents and cell lines

We have previously generated a human papillomavirus (HPV) E7-expressing tumorigenic cell line, TC-1 [17], and a firefly luciferase-expressing TC-1 cell line, TC-1-luc [2] (GenBank Accession LC456627.1). The H-2Db-restricted HPV16 E7aa49–57 peptide, RAHYNIVTF, was synthesized by Macromolecular Resources (Denver, CO) at a purity of ≥80%. PE-conjugated anti-mouse CD8a (clone 53.6.7), FITC-conjugated rat anti-mouse IFN-γ (clone XMG1.2) antibodies were purchased from BD Pharmingen (San Diego, CA). PE-conjugated, HPV16 E749–57 peptide loaded H-2Db tetramer was provided by NIAID tetramer core facility (Atlanta, GA). 3-BrPA, Benzo(a)pyrene (B(a)P, 99% pure), and tricaprylin were purchased from Sigma-Aldrich (St. Louis, MO).

### DNA constructs

We generated the DNA vaccine encoding calreticulin (CRT) and E7 through methods previously described [4]. We generated pcDNA3-CRT by first amplifying CRT by PCR using rabbit CRT cDNA as the template [20] and the primers 5′-CCGGTCTAGAATGCTGCTCCCTGTGCCGCT-3′ and 5′-CCGGGAATTCCAGCTCGTCCTTGGCCTGGC-3′. We then cloned the amplified product into the XbaI/EcoRI sites of the pcDNA3 vector (Invitrogen Corp.). To generate the pcDNA3-CRT/E7 vaccine (CRT/E7), we first amplified E7 with a set of primers (5′-GGGGAATTCATGGAGATACACCTA-3′ and 5′-GGTGGATCCTTGAGAACAGATGG-3′) and cloned into the EcoRI/BamHI site of pcDNA3-CRT. We confirmed the accuracy of our DNA constructs with DNA sequencing. We also previously tested the CRT/E7 DNA vaccine against a mock-DNA vaccine control [4].

### Tumor measurement

Tumor size was monitored by measuring the length (i.e., longest dimension) and width (i.e., shortest dimension) using dial calipers at 3-day intervals. Tumor volume was calculated by the following formula: *tumor diameter = 0.5 × (length + width)*.

### In vivo tumor treatment experiment

For in vivo tumor treatment, 1 × 10^5^ TC-1 tumor cells/mouse were subcutaneously (s.c.) injected into the left flank area of 6-to-8-week-old C57BL/6 mice on day 0. After 5 days, the mice were divided into 4 groups (5 mice/group), each receiving a different treatment regimen: Group 1 received no treatment after the TC-1 tumor challenge; Group 2 was treated with 3-BrPA by intraperitoneal (i.p.) injection (5 mg/kg of body weight) at 3-day intervals for a total 4 injections; Group 3 was immunized with the CRT/E7 DNA vaccine schedule at 3-day intervals for a total 4 injections; and Group 4 was both immunized with CRT/E7 and treated with 3-BrPA at 3-day intervals. Mice were monitored twice a week by inspection and palpation. 3-BrPA was prepared as suspensions in PBS. The CRT/E7 DNA vaccine, which has been described previously [15], was administered via gene gun in the amount of 2 μg/mouse beginning on day 5 at 3-day intervals for a total of 4 vaccinations.

### HPV16 E7-specific CD8+ T cell responses in tumor-bearing mice treated with 3-BrPA

Groups of C57BL/6 mice (5 mice/group) were challenged with TC-1 tumor cells and treated with 3-BrPA as described above. To detect HPV16 E7-specific CD8+ T cells in the spleen, splenocytes were harvested from the spleen one week after the last treatment. The cells were stained with FITC-conjugated anti-mouse CD8a (BD Pharmingen, San Diego, CA) and PE-conjugated HPV16 E7 aa49–57 peptide loaded H-2Db tetramer and acquired with FACSCalibur. To detect HPV16 E7-specific CD8+ T cells in the tumor, single cell suspensions were stimulated with HPV16 E7 aa49–57 peptide (1 μg/mL) in the presence of GolgiPlug (BD Pharmingen, San Diego, CA) overnight at 37 °C. The cells were then stained with PE-conjugated antimouse CD8a. After permeabilization and fixation, the cells were stained with FITC-conjugated anti-mouse IFN-γ followed by flow cytometry analysis.

### In vitro cytotoxic T cell assay

TC-1-luc tumor cells were added to 24-well plates (5 × 10^4^ cells/well) and incubated overnight at 37 °C. The cells were then treated with 3-BrPA (125 μM) for 24 h and were used as target cells. After washing with PBS, HPV16 E7-specific cytotoxic T lymphocytes (CTLs), generated as previously described [2], were added to each well except the control well and incubated at 37 °C for 4 h. Target cells incubated without CTLs served as a negative control. Luciferin (15 mg/mL/well) was added to the wells for optical imaging. The expression of luciferase was measured using the IVIS luminescence imaging system series 2000. Bioluminescence signals were acquired for 30 s.

### Immunofluorescence microscopy

TC-1 tumor cells were seeded to Lab-Tek chamber slide (5 × 10^4^ cells/slide) and then treated with 3-BrPA for 18 h. TC-1 tumor cells were then fixed with 4% formalin. Incubation with primary antibodies against CRT (Abcam, England) was carried out in PBS solution. Secondary Alexa 488 anti-rabbit IgG antibody were used (Life technologic, USA). All sections were counterstained with 4′,6-diamidino-2-phenylindole (DAPI, Life technologic, USA). Images were obtained using a confocal laser scanning microscope (LSM700, ZEISS, Germany).

### Statistical analyses

All experiments were replicated twice independently. All of our data are expressed as means ± standard error (SE) and are representative of at least two independent experiments. Unless indicated, the statistical significance of difference was assessed by two-tailed Student’s t-tests using SPSS version 20.0. The level of significance was set at *p* < 0.05.

## Results

### 3-bromopyruvate generates potent antitumor effects in TC-1 tumor-bearing mice

**Fig. 1:**
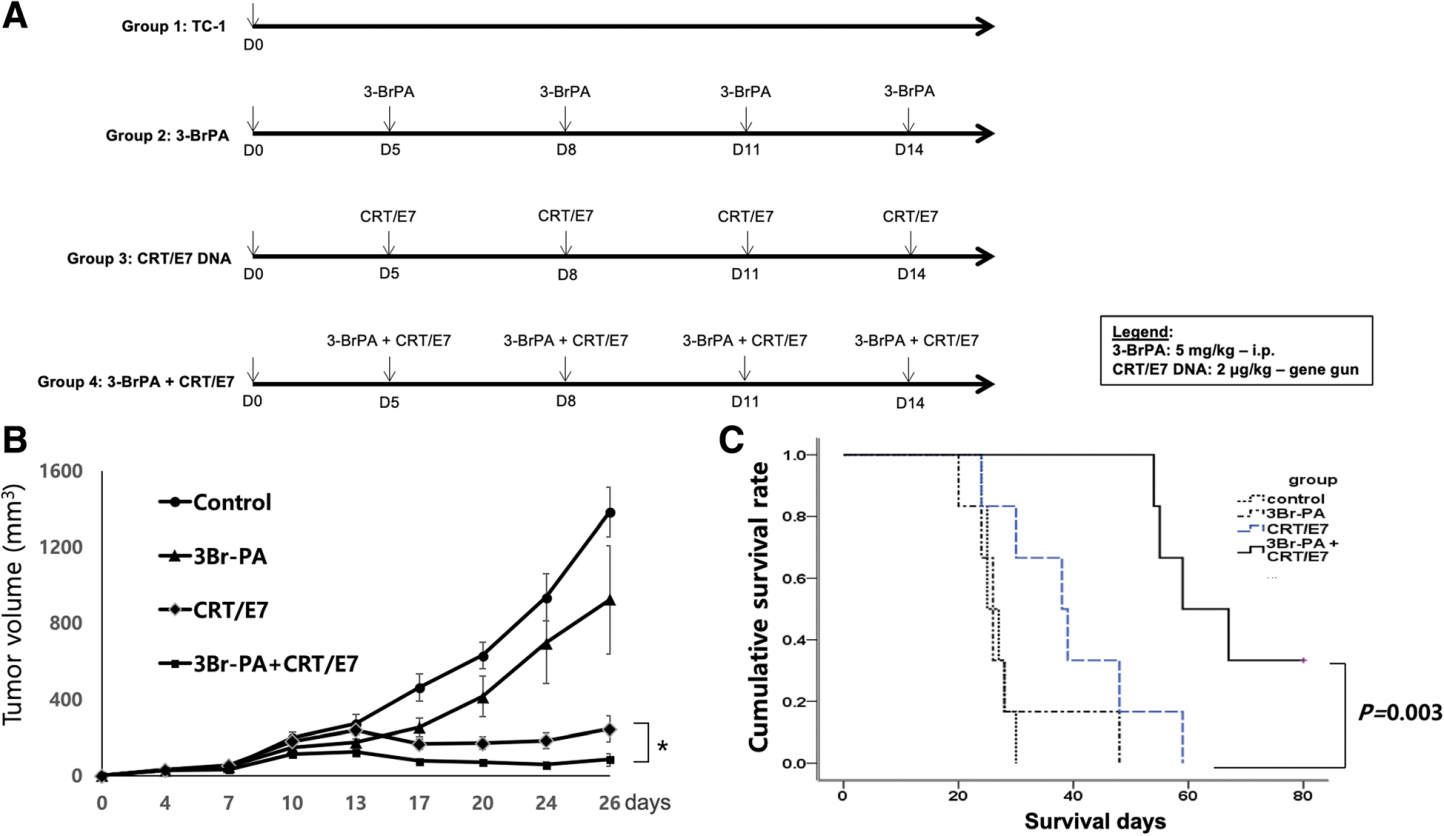
In vivo tumor treatment experiments. **a** Schematic illustration of the treatment schedule. Groups of C57BL/6 mice (5 mice/group) were s.c. challenged with 1 × 10^5^ TC-1 tumor cells/mouse on day 0. TC-1 tumor-bearing mice were treated with the CRT/E7 DNA vaccine and/or 3-BrPA as indicated in the timeline. **b** Line graph depicting the tumor volume in TC-1 tumor-bearing mice treated with the different treatment regimens. Each plot point represents the mean tumor volume of each group; the standard error is indicated by the bars. *p < 0.05. **c** Kaplan-Meier survival analysis of TC-1 tumor-bearing mice treated with the different treatment regimens

We first characterized the antitumor effects of the ER stress agent 3-BrPA, alone or in combination with the CRT/E7 DNA vaccine against the E7-expressing TC-1 tumor model in C57BL/6 mice. The mice were treated according to the regimens outlined in Fig. 1a. The various treatment strategies were initiated 5 days after initial challenge of TC-1 cells. 3-BrPA was injected i.p. at 3-day intervals. The CRT/E7 DNA vaccine was administered at 3-day intervals from day 5 to day 11 by intramuscular (i.m.) injection. As shown in Fig. 1b, mice treated with 3-PrPA and the CRT/E7 vaccine had lower tumor volumes than mice treated with either 3-BrPA or CRT/E7 alone. Furthermore, survival rates of mice treated with the combination of 3-BrPA and CRT/E7 DNA vaccine significantly increased compared to any other treatment regimen (Fig. 1c). These data suggest that a combinatorial treatment with 3-BrPA and the CRT/E7 DNA vaccine elicits synergistic antitumor effects in TC-1 tumor-bearing mice.

### TC-1 tumor-bearing mice receiving both 3- bromopyruvate and CRT/E7 DNA generated greater numbers of E7-specific CD8+ T cells

**Fig. 2:**
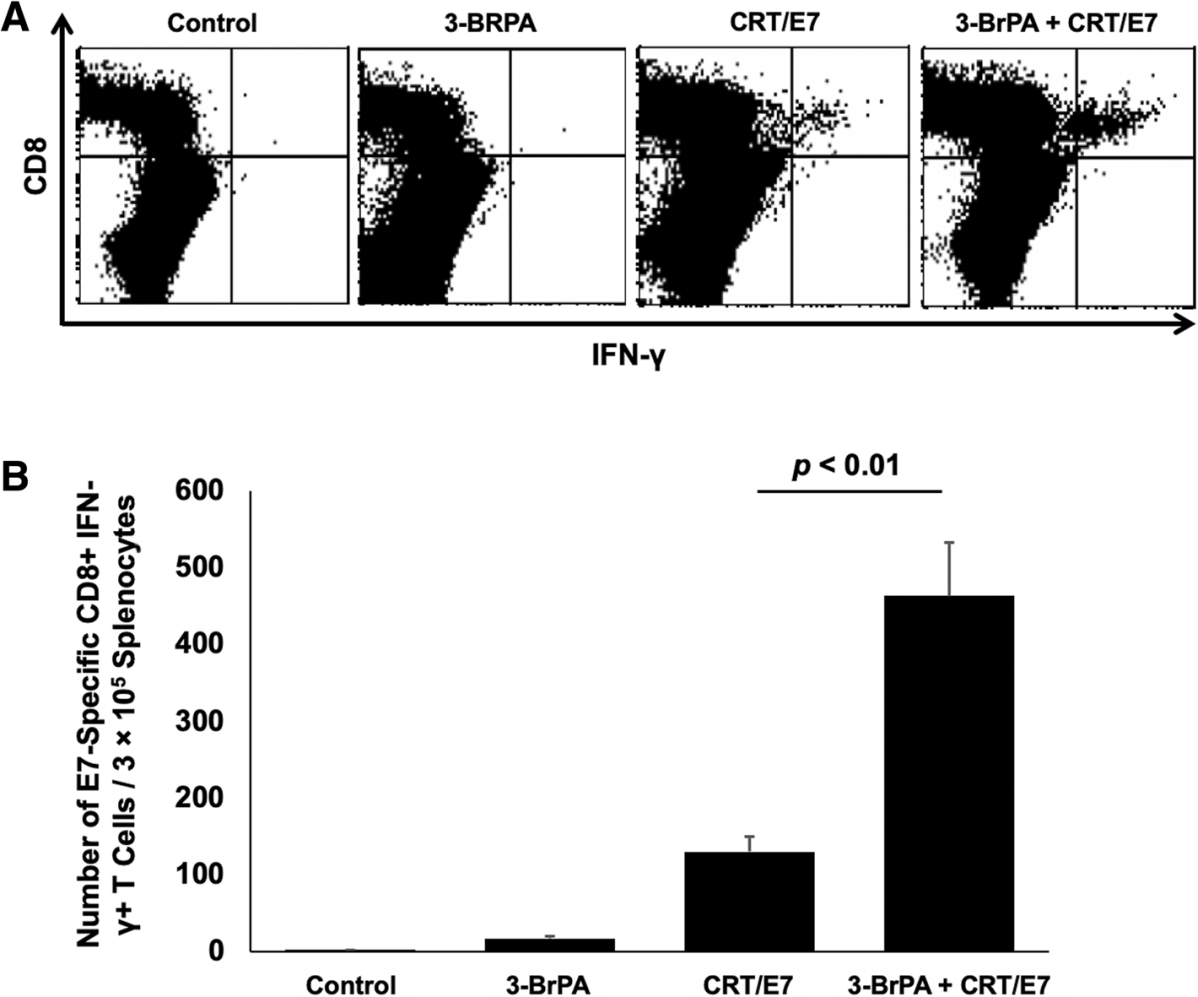
Characterization of E7-specific CD8+ T cells. Intracellular cytokine staining and flow cytometric analysis to determine the number of IFN-γ–secreting E7-specific CD8+ T cells in tumor-bearing mice treated with 3-BrPA and/or CRT/E7. On day 21, splenocytes from the treated TC-1 tumor-bearing mice were harvested and incubated with the E7 peptide overnight. Among complete splenocytes, E7-specific CD8+ T cells were quantified using intracellular staining for IFN-γ, followed by flow cytometry analysis. **a** Representative flow cytometric analyses data shown. **b** Bar graph depicting the number of E7-specific IFN-γ-producing CD8+ T cells per 3 × 10^5^ splenocytes. Each column represents the mean T cell count of each group; the standard deviation is indicated by the bars. Data is represented by the mean ± SD of three independent experiments

Intracellular cytokine staining and flow cytometric analyses were used to determine the number of IFN-γ-secreting E7-specific CD8+ T cells in tumor-bearing mice treated with 3-BrPA and/or CRT/E7. On day 21 after initial tumor challenge, splenocytes were harvested, isolated, and characterized for E7-specific CD8+ T cells. The combination treatment of 3-BrPA and CRT/E7 generated the highest proportion of E7-specific CD8+ T cells among all treatment groups (Fig. 2). Our data shows that 3-BrPA treatment enhances the generation of E7-specific CD8+ T cells generated by the CRT/E7 DNA vaccine.

### 3-bromopyruvate increases ER stress markers in TC-1 cells, and elevates the expression of CRT in TC-1 cells

**Fig. 3:**
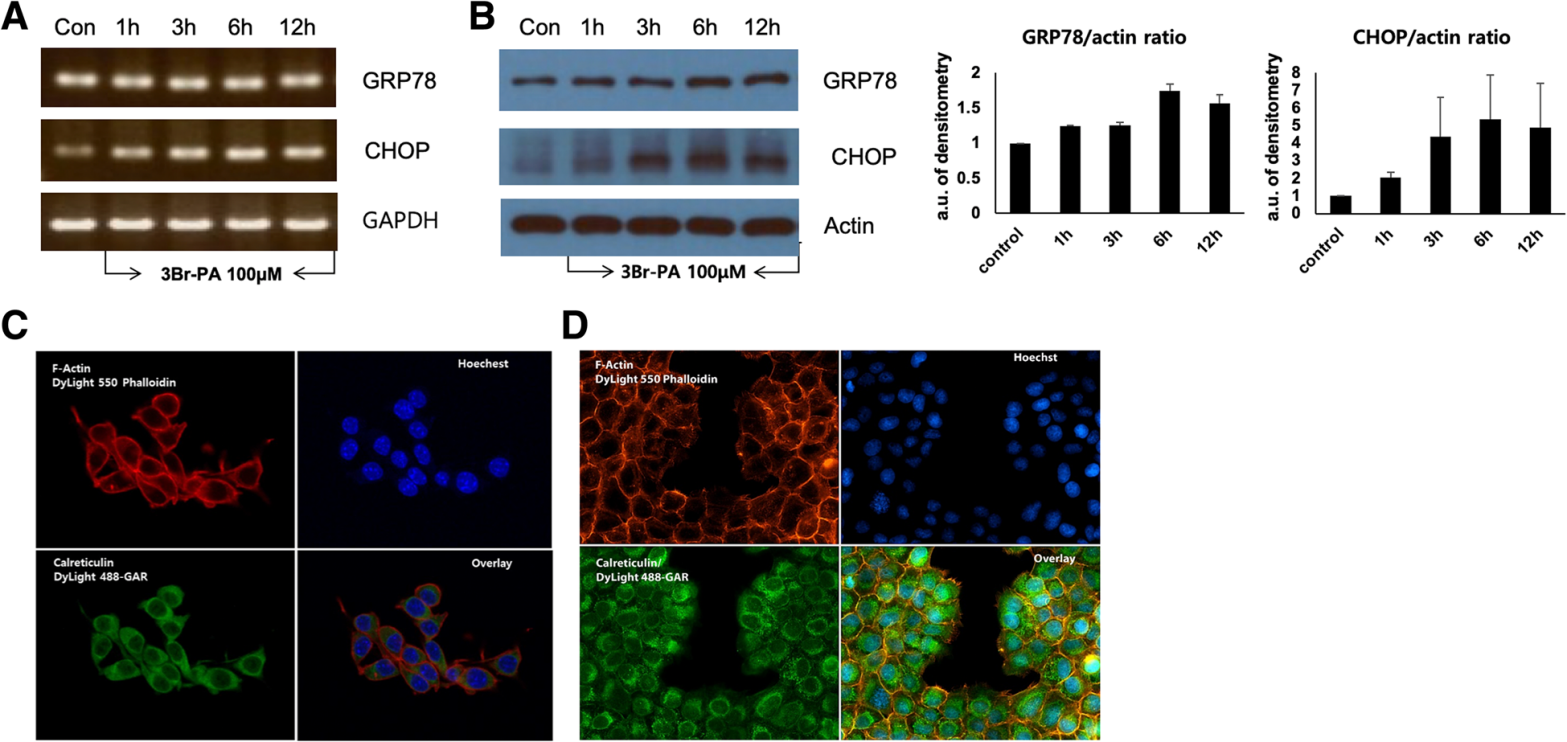
3-bromopyruvate increased the ER stress markers in TC-1 cells. **a** Representative RT-PCR of GRP78 and CHOP mRNA expression (25 cycles of PCR). **b** Representative Western blot of GRP78 and CHOP in TC-1 cells. GRP78 and CHOP activities were calculated as ratios with respect to actin. **c**, **d** Representative confocal laser immunofluorescence photomicrographs of (**c**) control TC-1 cells and (**d**) 3-BrPA-treated TC-1 cells. 3-BrPA increased the CRT on the surface membrane of TC-1 cells

We then investigated the effect of 3-BrPA on ER stress and the expression of CRT in TC-1 cells. The results showed that the expression of ER stress markers and CRT were significantly increased in 3-BrPA-treated TC-1 cells. To evaluate the intensity of ER stress, expression levels of ER stress markers CCAAT-enhancer-binding protein homologous protein (CHOP) and glucose regular protein 78 (GRP78) in TC-1 cells were determined through RT-PCR and Western blots. The results revealed that mRNA levels of GRP78 and CHOP in 3-BrPA-treated TC-1 cells were gradually increased (Fig. 3a). Western blot analyses revealed that protein levels of GRP78 and CHOP in TC-1 cells were also increased from 6 h and 3 h, respectively (Fig. 3b). We also measured the translocation of CRT after treatment of 3-BrPA using immunofluorescence staining and confocal microscopy. Confocal microscopic analyses revealed that 3-BrPA-treated TC-1 cells showed immunofluorescence of CRT in TC-1 cells predominantly in cell membrane areas compared with 3-BrPA-untreated TC-1 cells (Fig. 3c, d).

### 3-bromopyruvate increases E7-specific T cell cytotoxicity

**Fig. 4:**
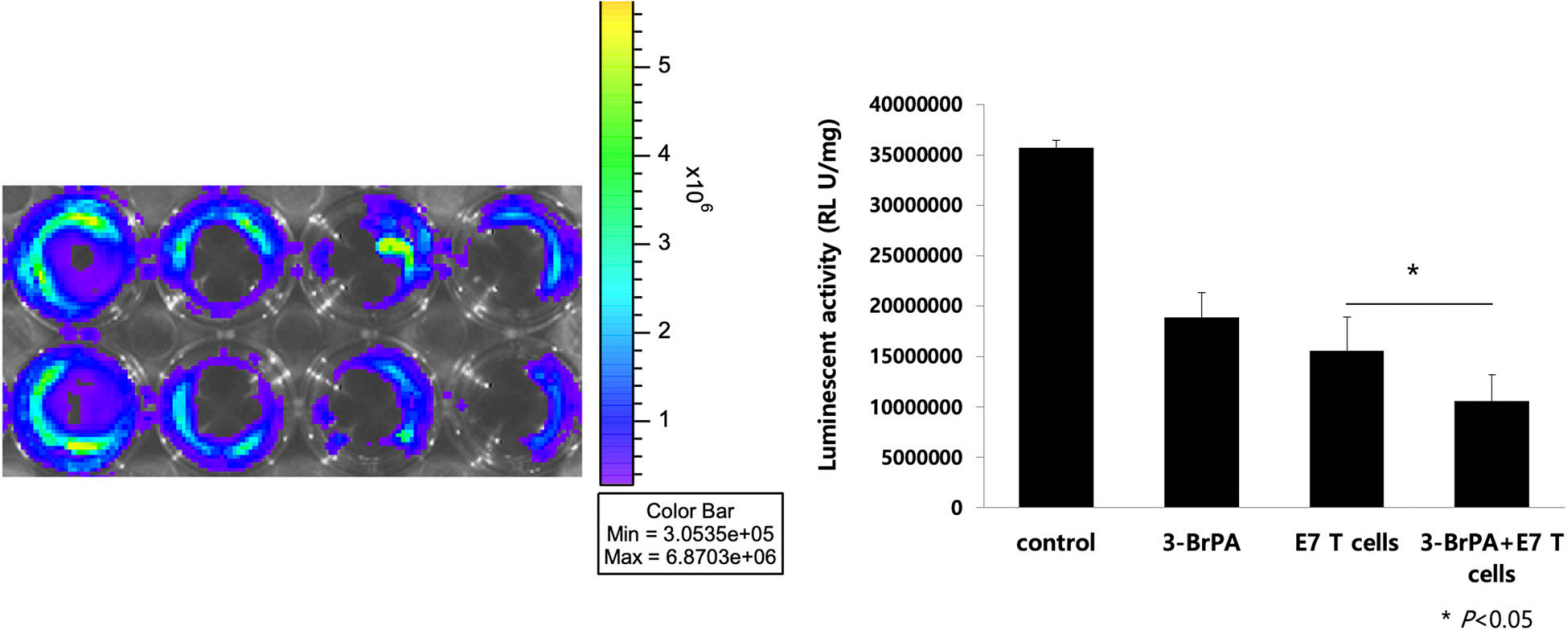
3-bromopyruvate increased the cytotoxicity of E7-specific T in vitro. Luciferase-expressing TC-1 tumor cells were seeded in 24-well plates (5 × 10^4^ cells/well). The following day, the medium was replaced with fresh medium containing 3-BrPA. 24 h later, the media was replaced again, removing 3-BrPA, and 1 × 10^6^ E7-specific CTLs were added to each well. The degree of CTL-mediated killing of the tumor cells is indicated by the decrease of luminescence activity and measured with the IVIS Luminescence Imaging System Series 200 (bioluminescence signals were acquired for 3 mins). Data is represented by mean ± SD of three independent experiments (*p < 0.05)

In order to determine whether 3-BrPA enhanced the danger signal through CHOP and could increase the susceptibility of TC-1 tumor cells to killing by E7-specific CD8+ T cells, we incubated E7-specific CD8+ T cells with TC-1-luc tumor cells with or without pre-treatment with 3-BrPA. Untreated TC-1-luc tumor cells were included as a control. As shown in Fig. 4, TC-1-luc cells treated with 3-BrPA and incubated with E7-specific CD8+ T cells had a much greater decrease in luminescence, indicating greater cytotoxicity, than TC-1-luc cells treated with either 3-BrPA or incubated with E7-specific CD8+ T cells alone. Taken together, our data suggest that TC-1 tumor cells treated with 3-BrPA are able to enhance immunogenic cell death, resulting in their increased susceptibility to killing by E7-specific CD8+ T cells.

## Discussion

In our study, we found that 3-BrPA treatment led to the upregulation of ER stress in TC-1 tumor cells. When cancer cells die, they undergo immunogenic apoptosis characterized by surface-exposed CRT and induced E7-specific CD8+ CTL cytotoxicity. We also found that while 3-BrPA elicited antitumor effects in TC-1 tumor-bearing mice, the combination of 3-BrPA and CRT/E7 created a synergistic effect leading to the generation of significantly more E7-specific CD8+ T cells in the spleen compared to treatment with either treatment alone.

Recently, several DAMPs have been identified as crucial for immunogenic apoptosis. These include surface CRT, surface heat shock protein 90 (HSP90), and secreted ATP [6, 27]. The ER is a specialized organelle that plays a central role in the biosynthesis, correct protein folding, and post-translational modifications of secretory and membrane proteins [10]. However, when the process of protein synthesis and protein folding is out of balance, unfolded and misfolded proteins are accumulated in the ER lumen, which is referred to as ER stress [29]. Cells initiate an adaptive response known as the “unfolded protein response” (UPR) to maintain homeostasis of ER function against an excess of ER stress. ER stress increases in expression of GRP78, a prominent ER-resident chaperone, and CHOP, an apoptotic transcriptional factor [5, 12]. UPR activation functions primarily to protect the cell from ER stress. However, continuous ER stress beyond the threshold of adaptation can trigger apoptosis [7]. Severe ER stress can induce a switch in UPR signaling from pro-survival to pro-apoptotic pathways, which involve the induction of CHOP and the activation of pro-apoptotic kinases such as apoptosis signal regulating kinase 1 (ASK1) and c-Jun-NH_2_-terminal kinase (JNK). In this experiment, 3-BrPA treatment increased the expression of GRP78 and CHOP in TC-1 cells and increased the expression of CRT on TC-1 cell surfaces. CRT can control the ER to prevent protein misfolding and the aggregation and secretion of incompletely translated major histocompatibility complex I molecules [34]. When apoptosis is induced in tumor cells, CRT is quickly translocated from the ER to the surfaces of cell membranes, leading to immunogenic cell death of tumor cell. When we knocked out CRT, immune responses such as the activation of dendritic cells (DCs) by apoptotic cells also disappeared [16]. In addition to 3-BrPA, ER stress-inducing agents such as bortezomib, oxaliplatin, and oncolytic virus also induce ER stress to induce immunogenic cell death by expressing DAMP signals (e.g. calreticulin) on the cell surface, thereby activating the immune system of the tumor microenvironment [23]. Several biochemical assays have also been developed in the past decade to measure the induction of ER stress, surface exposure of ER/cytosolic chaperones CRT and release of ATP or HMGB1 as markers of immunogenic cell death (ICD) induction. However, none of these assays, even in combination, could discriminate with certainty between ICD and non-ICD inducers. Therefore, ICD evaluation still requires systematic analysis of the generation of an antitumor adaptive immune response. Although we did not check the expression of ER stress markers (e.g. CHOP, GRP78) in the mouse tumor model, we found that the E7-specific CD8 + T cells increased in the spleen and increased ER stress molecules in vitro.

In our experiment, we demonstrate that 3-BrPA may be able to induce the translocation of CRT to the surfaces of tumor cell membranes as a result of ER stress and activate the “eat me” pathway [19, 26, 33]. This “eat me” pathway stimulates the phagocytosis of DCs, which are important antigen presenting cells that initiate T cell-mediated immune responses. Upon recognition and presentation by DCs, tumor antigens trigger T cell-dependent antitumor immune response [14]. The addition of an E7-specific DNA vaccine to these activated DCs further increases the immune activity of DCs and promotes further T cell differentiation. Furthermore, UPR events have been implicated in immune responses, including T cell differentiation and terminal B cell differentiation [9, 25]. These processes eventually promote the differentiation of E7-specific T cells and consequently induces killing of E7-expressing TC-1 cells, resulting in the delay of tumor growth. Those results showed that 3-BrPA and E7-specific DNA vaccine combination therapy can induce specifically sensitized CD8+ T lymphocytes with reconstructed immune pattern within the tumor.

## Conclusions

In summary, the results of our study suggest that treatment of tumor-bearing mice with 3-BrPA may create potentially potent immune-mediated therapeutic antitumor effects not only through enhanced tumor-specific immunity, but also through an increased susceptibility of tumor cells to antigen-specific CD8+ T cell-mediated killing. Thus, our study serves as an important foundation for the future clinical application of 3-BrPA in cancer treatment and immunotherapy.

## Data Availability

All data recorded for this study was performed at the Johns Hopkins Medical Institutes.

## Abbreviations

3-BrPA: 3-bromopyruvate
ASK1: Apoptosis signal regulating kinase 1
CHOP: CCAAT-enhancer-binding protein homologous protein
CREBH: Cyclic-AMP-responsive-element-binding protein H
CRT: Calreticulin
CRT/E7: pcDNA3-CRT/E7 vaccine
CTL: Cytotoxic T lymphocytes
DAMP: Damage-associated molecular pattern
DC: Dendritic cell
ER: Endoplasmic reticulum
GRP78: Glucose regular protein 78
HPV: Human papillomavirus
HSP90: Heat shock protein 90
i.m.: Intramuscular
i.p.: Intraperitoneal
JNK: c-Jun-NH_2_-terminal kinase
s.c.: subcutaneous
UPR: Unfolded protein response
